# Supplementary material for: Quality of life in Arab children with congenital heart disease
Source: PLoS One. 2024 Jan 18;19(1):e0290306. doi: 10.1371/journal.pone.0290306 (PMC10796050; doi:10.1371/journal.pone.0290306)
Supplement: Attachment — Submitted filename: Review comments on Manuscript Number.doc [file pone.0290306.s001.doc]

**Review comments on Manuscript Number: PONE-D-23-03793. Entitled " Quality of Life in Arab Children and Adolescents with Congenital Heart Disease"**

Overall, the idea of research is very interesting to be studied nowadays and paper is coherently developed. However, there are some comments and suggestions.

**Title**

- Well structured

**Abstract**

- Well structured

**Introduction**

- Well structured

**Materials and methods**

- The participants sections is recommended to be moved after study design

**Statistical analysis**

- Well structured
- The subtitles (Aim One: Compare the levels of QoL from parents’ perspectives to those of their children’s perspectives/ Aim Two: assess determinants of QoL in children and adolescents with CHD). You may replace by (levels of QoL from parents’ perspectives to those of their children’s perspectives/ determinants of QoL in children and adolescents with CHD)

**Discussion**

- Well structured
